# Habitat suitability and demography, a time‐dependent relationship

**DOI:** 10.1002/ece3.2821

**Published:** 2017-03-02

**Authors:** Léo Bacon, Yves Hingrat, Frédéric Jiguet, Anne‐Christine Monnet, François Sarrazin, Alexandre Robert

**Affiliations:** ^1^ Emirates Center for Wildlife PropagationMissourMorocco; ^2^ Centre d'Ecologie et des Sciences de la COnservation (CESCO UMR 7204)Museum National d'Histoire NaturelleParisFrance; ^3^ RENECO International Wildlife Consultants LLCAbu DhabiUAE

**Keywords:** Biotic interactions, nest survival, niche modeling, niche switch, seasonal dynamics

## Abstract

The habitat suitability index, which reflects spatial variability in species occurrence probability, has been shown to exhibit various contrasting relationships with local demographic performances (survival, productivity) in several species. One proposed explanation for these discrepancies is that the link between the habitat suitability index and demography is influenced by density‐dependent, temporally variable processes. Based on the survival rates of more than 3,000 nests monitored over 12 years in the North African Houbara Bustard, we investigated whether the habitat suitability index computed over the species breeding range is related to nest survival throughout the breeding season, accounting for variation in meteorological conditions. We found that the relationship between the habitat suitability index and nest survival progressively changes along the breeding season and that this intra‐annual variation is consistent between years. Our results support the hypothesis that variation in space use occurs intra‐annually and that biotic interactions throughout the breeding season strongly influence the habitat suitability index–demography relationship.

## 1 Introduction

The habitat suitability index (HSI) is a probability of species presence inferred from ecological niche modeling, by relating the occurrence of a species at a given location to environmental features (Guisan & Thuiller, 2005). The output of species distribution models based on such niche modeling is considered as a measure of the suitability of environmental features for the occurrence of the target species (VanDerWal et al., 2009). Thus, if habitat selection is adaptive, habitat attractiveness should match demographic performance (Battin, 2004; Hutchinson, 1957) and the HSI should be related not only to the probability of occurrence, but also to other key parameters of populations.

Recent studies have attempted to link the HSI to various demographic parameters, such as survival (Monnet, Hardouin, Robert, Hingrat, & Jiguet, 2015; Unglaub, Steinfartz, Drechsler, & Schmidt, 2015) and reproductive parameters (Brambilla & Ficetola, 2012; Pellissier et al., 2013; Thuiller et al., 2014; Unglaub et al., 2015). Results from these studies exhibited various contrasting relationships, including positive, negative, and null correlations. Three general explanations have been used to rationalize these complex and inconsistent relationships. First, some empirical evidence suggests that environmental conditions favoring both species presence and the demographic performance of that given species can be distinct from those favoring only its presence (see an example in an alien population in Ficetola, Miaud, Pompanon, & Taberlet, 2008). For example, ecological theory regarding source–sink dynamics (Pulliam & Danielson, 1991) and ecological traps (Kristan, 2003) indicates that, in certain species, some individuals may be located outside the bounds of the species niche (Bateman, VanDerWal, & Johnson, 2012; Schurr et al., 2012; Titeux, Dufrene, Radoux, Hirzel, & Defourny, 2007). Second, high HSI values might reflect high local densities (Tôrres et al., 2012), and various ecological processes can be modulated by density (Oliver et al., 2012), such as intra‐ and interspecific competitions or predation (Fernández‐Bellon, Cortés‐Avizanda, Arenas, & Donàzar, 2016; Guisan & Thuiller, 2005; Gunnarsson & Elmberg, 2008; Thuiller et al., 2014), which can in turn produce unexpected and complex relationships between the HSI and demographic rates. A third proposed explanation is that the link between the HSI and demography is temporally variable (Wisz et al., 2013). More specifically, the influence or correlation between some environmental features and demographic rates can vary within and between years due to factors such as the phenology of the species, seasonal and interannual variations in biotic interactions, demographic heterogeneity, meteorological fluctuations, or climate change (Yang & Rudolf, 2010). For example, many studies have documented that biotic interactions are not static (Brooker, 2006; Callaway et al., 2002; Forchhammer, Post, Berg, Høye, & Schmidt, 2005; Gilman, Urban, Tewksbury, Gilchrist, & Holt, 2010), and static species distribution models may fail to recognize the effects of species interactions if temporal variation is not considered (see Bateman et al., 2012). Other results indicate that this is also true for abiotic factors, such as short‐term meteorological variation (Reside, VanDerWal, Kutt, & Perkins, 2010). Finally, the link between the HSI and demography can change, for example, at the intra‐annual scale, through niche dynamic processes, for example, a switch of the niche (i.e., space use) between the breeding and nonbreeding seasons in species with seasonal breeding behaviors (Hayes, Cryan, & Wunder, 2015; Nakazawa, Peterson, Martinez‐Meyer, & Navarro‐Siguenza, 2004; Yannic et al., 2014). Any subsequent change in local population size or density can in turn impact demographic rates (Brook & Bradshaw, 2006; Fernández‐Bellon et al., 2016; Frederiksen & Bregnballe, 2000).

Here, we aim to examine the relationship between the HSI and demography at both interannual and intra‐annual scales, with special emphasis on the dynamic aspect of this relationship. Using data collected from a longitudinal nest survey in a bird species, the North African Houbara Bustard (*Chlamydotis undulata undulata*) (hereafter “Houbara”), we explored whether the breeding performance (estimated by the daily nest survival rate) is related to the HSI computed from independent data, which represents a suitability index of presence, over the breeding range of the species and over several years, depending on meteorological conditions, as well as throughout the breeding season.

Previous research on Houbara and its breeding environment indicates that (1) there are significant biotic and abiotic changes in the breeding environment of birds along the breeding season (ranging from February to June), including meteorological changes (Warner, 2004) and changes in intraspecific interactions (Hingrat, Saint Jalme, Ysnel, Le Nuz, & Lacroix, 2007), as well as in interspecific interactions (Bourass et al., 2012; Hingrat, Ysnel et al. 2007); (2) young, inexperienced females tend to breed later in the season compared to older females (Bacon, 2013), which is likely to generate some demographic heterogeneity along the season; (3) interannual variation in meteorological conditions can affect demographic rates (see Hardouin et al., 2014 for survival); (4) in captive‐bred individuals released in the wild, survival is related to changes in local HSI values along postrelease movements (Monnet et al., 2015). Birds moving to sites with lower HSI values than the HSI values of their release site have a reduced survival probability, while individuals moving to sites with higher HSI have increased survival probability. However, individuals with the highest HSI gain do not have the highest survival probability. Monnet et al., 2015 set forward the hypothesis that higher densities in high HSI sites may enhance deleterious ecological processes such as intraspecific competition.

Based on these elements, we expect a negative relationship between the HSI of the Houbara breeding range and the daily nest survival rate, potentially caused by density‐dependent effects that could be magnified in years with low resource availability (e.g., low precipitation) or during the peak of the breeding season (in April) when breeding activity and competition are the highest.

## 2 Methods

### 2.1 Study species and study areas

The Houbara is a medium‐sized, nonmigratory bustard inhabiting semidesert habitats from North Mauritania to Egypt. It is a ground‐nesting, gyneparental incubating species which breeds from mid‐February to mid‐June. Females typically lay 2–3 eggs per clutch, which they incubate for about 23 days (Gaucher, 1995). Houbara chicks are nidifugous.

**Figure 1:**
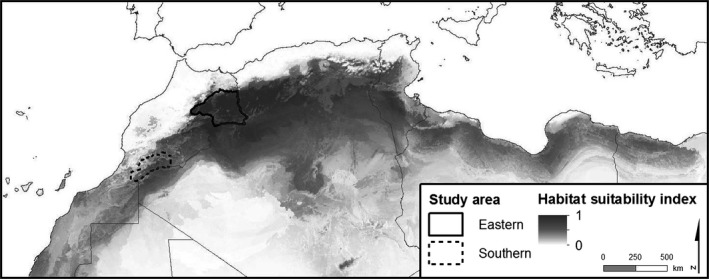
Locations of the study areas within the habitat suitability index (HSI) gradient of the North African Houbara Bustard (*Chlamydotis undulata undulata*) breeding range (Monnet et al., 2015)

Our study was conducted between 2003 and 2014 in two study areas in southern and eastern Morocco totaling 75,000 km² (Figure 1). These two study areas are part of the Emirates Center for Wildlife Propagation (ECWP) intervention areas. In these intervention areas, the Houbara population is reinforced through regular releases of captive‐bred individuals (Chargé et al., 2014; Hardouin et al., 2014).

### 2.2 Data collection

#### 2.2.1 Demographic data

Between 2003 and 2014, 3,108 nests were located from a collaborative survey involving local shepherds and from the monitoring of females fitted with radio and satellite transmitters (see Appendix A1, Table A1.1, Figure A1.1 in Appendix S1). For most nests, visits occurred every 1–10 days (first and ninth centile of visit interval distribution). Visits were more frequent at the end of the incubation stage to obtain better accuracy on the dates of hatching. In addition, in 2013 and 2014, camera traps were deployed on some of the monitored nests (*n* = 231), to obtain a continuous survey effort at these nests. Nests equipped with camera traps were visited every 3.7–8 days (first and ninth centile of visit intervals distribution, see Appendix A1, Table A1.2, Figure A1.2 in Appendix S1). Nest monitoring effort was constant between years (see details in Appendix A1 in Appendix S1). Nest failure was considered to have occurred for a given visit interval if the clutch disappeared without signs of hatching (Mabee, 1997) or was abandoned.

#### 2.2.2 Spatial covariate: the HSI

We extracted the value of the habitat suitability index (HSI) of the Houbara breeding range, computed by Monnet et al. (2015) (Figure 1), at each nest location. This index is based on occurrences (presence and pseudo‐absence) related to spatial variation in the environment, including both land cover and climate variables (see Appendix A2 in Appendix S1). Considering the large decline of the species these last decades, Monnet et al. (2015) used both historical and recent occurrence data to model the ecological niche of Houbara. Historical data were randomly sampled in the historical breeding range of the species (Goriup, 1997) and completed by data collected in Morocco, Tunisia, and Algeria between 1997 and 2012 from monitoring wild adult birds with satellite and VHF transmitters recorded during the breeding period (from February to May, see Monnet et al., 2015 for more details). Monnet et al. (2015) summarized occurrence and environmental data at a 2.5 arc‐minutes grid (approximatively 4.5 × 4.5 km). They performed a consensus approach using the BIOMOD computer platform (Thuiller, Lafourcad, Engler, & Araujo, 2009). All models were evaluated by computing the area under the curve (AUC) and showed high predictive performance (AUC > 0.85, Araújo, Pearson, Thuiller, & Erhard, 2005). Following one of the best consensus methods identified by Marmion, Parviainen, Luoto, Heikkinen, and Thuiller (2009), the final HSI was obtained by calculating the mean value of the predictions across the runs of seven modeling techniques. Further details on occurrence data and niche modeling techniques can be found in Ref. Monnet et al. (2015).

#### 2.2.3 Temporal covariates

In semidesert habitats, meteorological conditions from past seasons have a major influence on seasonal productivity of the habitat (e.g., precipitation legacies; Ozenda, 2004; Reichmann, Sala, & Peters, 2013; Sala, Gherardi, Reichmann, Jobbágy, & Peters, 2012) and potentially habitat selection within Houbara populations. Therefore, variability in meteorological conditions before the breeding season may affect the relevance of the static HSI during breeding seasons. We thus assigned for all nests from the same study area the average precipitation value (*Prec*, in mm/hr) and average surface air temperature value (*Temp*, in °C) from the previous year, obtained from the Global Land Data Assimilation System (GLDAS v001, Rodell et al., 2004).

To account for potential variation in the daily nest survival rate within the nesting season, we included the date in the nesting season (*Date*, as julian days) as an intraseasonal explanatory covariate, as it may represent a gradient of meteorological conditions (Warner, 2004), habitat selection (Hingrat, Saint Jalme et al. 2007), and species interaction (Hingrat, Ysnel et al. 2007) within the breeding season.

All covariates were rescaled, being centralized and standardized by two times the standard deviation (Gelman, 2008). Because our main objective was to assess the relationship between daily nest survival rate and HSI (i.e., a spatial index of quality, which already carries information on average local climatic conditions), the rescaling of meteorological data (meteorological anomalies) was done independently for the two study areas, which allowed us to increase their interannual variation at the expense of the spatial variation. Thus, our data allowed us to assess the combined effects of spatial (HSI: one unique value for each nest location over the study period) and temporal (meteorological anomalies, date within the breeding season) variations on demography. Correlations between the rescaled covariates were all weak (Pearson correlation tests |*r*| < 0.3).

### 2.3 Nest survival and statistical analysis

We assessed the effects of spatial and temporal covariates on the daily nest survival rate (probability of a live nest to not fail in 1 day), using the logistic exposure approach from Shaffer (2004). This generalized linear method incorporates a logit link function that performs with various exposure periods between nest visits (Shaffer, 2004). It attempts to overcome biases associated with other estimator techniques that capture apparent nest survival (proportion of nests with at least one egg hatching). For hatching nests, the exposure period stops with the first egg hatching. This approach does not require the exact day of nest failure but the interval within which the failure occurred. We focused on the daily survival rate as we considered it to be the most relevant proxy of breeding performance to link to large‐scale environmental features. The Houbara has nidifugous chicks, and data on chick survival or total reproductive output are scarce. In addition, nests are fixed entities and the link between HSI and nest survival cannot be mitigated by individual movements of chicks. Therefore, we preferred to focus on an easily acquired breeding parameter from which we could have the highest range of HSI sites.

We first examined the relationship between the daily nest survival rate and covariates by developing a set of candidate models including all combinations of additive effects. This first round of model selection allowed us to assess the global relationship between the daily nest survival rate and the HSI while controlling for other potential effects, but intentionally neglecting potential temporal variation in the daily nest survival rate–HSI relationship. As a second step, we specifically investigated whether the daily nest survival rate–HSI relationship could temporally vary within the breeding season and/or be driven by meteorological variation among years by developing a set of candidate models including additive effects and the first order interactions *HSI *× *Date*,* HSI *× *Prec,* and *HSI *× *Temp*. We partitioned our analysis into two separate steps (additive effects only and additive plus interaction effects) because the use of model averaging techniques to compare additive effects is irrelevant when interaction terms are present. The year and study area were incorporated as random effects in all models. We performed a full multimodel inference analysis (Burnham & Anderson, 2002) in each of these two steps (only the starting full model differed between the two steps).

We ranked models using the Akaike information criterion corrected for small sample size (AICc). For each step, we estimated robust model‐averaged coefficients (β) from the set of best models (ΔAICc < 2; Burnham & Anderson, 2002), based on their AICc weights (the relative likelihood of candidate models based on their ΔAICc). We assumed significance of β for the covariates if their 95% unconditional confidence intervals (CIs) did not include zero. All analyses were conducted using R 3.0.2 (R Development Core Team, 2013) and the packages lme4 1.1.7 (Bates, Martin, Bolker, & Walker, 2015) and MuMIn 1.13.4 (Barton, 2015). The logistic exposure link function implemented in generalized linear mixed models was obtained from the R package nest survival (Herzog, 2009).

## 3 Results

From the sample of 3,108 monitored nests, a total of 1,391 (45%) failed. The values of the habitat suitability index (HSI) at the nest locations ranged from 0.10 to 0.91, with a median at 0.86 (see Appendix B1 in Appendix S2).

From the first set of models (focusing on additive effects, see details on model ranking in Appendix B2.1 in Appendix S2), the analysis indicated that the daily nest survival rate decreased with the progression of the breeding season, but no global effect of the HSI on nest survival was observed (Table 1).

**Table 1:** Model‐averaged coefficients and 95% confidence intervals (CIs) from the set of best models (ΔAICc < 2) for the analysis of the additive effects of spatial and temporal covariates on the daily nest survival rate

Covariate	Coefficients	Lower CI	Upper CI	Relative importance
**Date**	−**0.38**	−**0.49**	−**0.27**	**1**
HSI	−0.11	−0.22	0.01	0.69
Prec	0.09	−0.02	0.21	0.52
Temp	−0.03	−0.13	0.07	0.28

The relative importance of the covariate is the sum of AICc weights of the models where the covariate occurs. Random effects for the year and study area are fitted for all models. Coefficients are in bold where CIs do not include zero.

**Figure 2:**
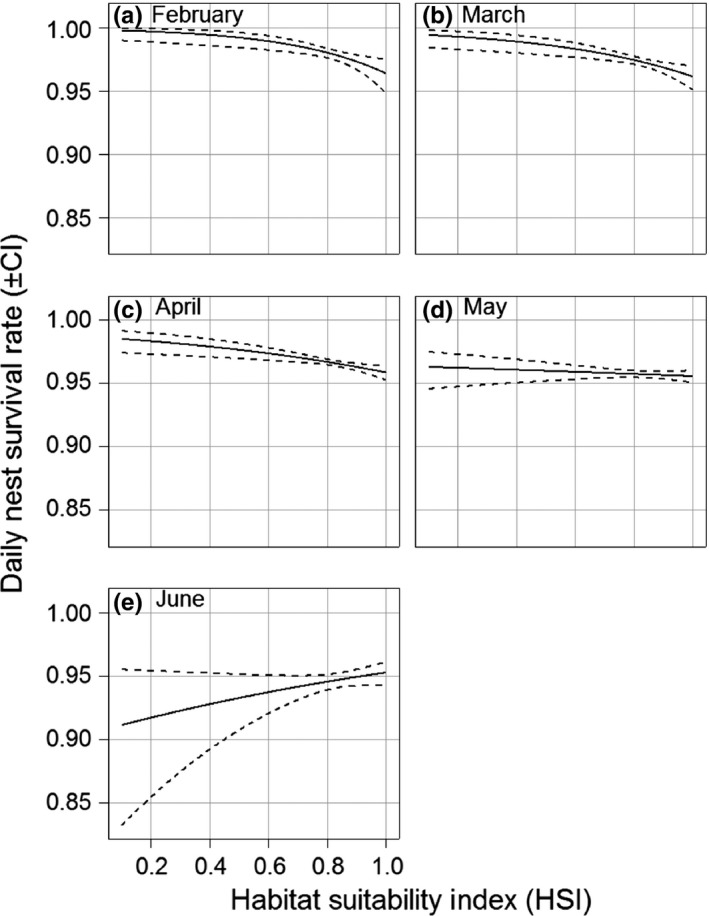
Relationship between the daily nest survival rate (±95% CI) and the habitat suitability index (HSI) for each month of the breeding season. (a) February, (b) March, (c) April, (d) May, and (e) June

The second set of models (models with interactions, see details in Appendix B2.2 in Appendix S2) revealed a strong positive interaction between *HSI* and *Date* (Table 2). The daily nest survival rate was negatively related to the HSI early in the nesting season, and this relationship became positive by the end of the season (Figure 2). No significant interactions between HSI and meteorological anomalies were found.

**Table 2:** Model‐averaged coefficients and 95% confidence intervals (CIs) from the set of best models (ΔAICc < 2) for the analysis of habitat suitability interactions on the daily nest survival rate

Covariate	Coefficients	Lower CI	Upper CI	Relative importance
**Date**	−**0.35**	−**0.46**	−**0.23**	**1**
**HSI**	−**0.18**	−**0.30**	−**0.05**	**0.98**
**Date × HSI**	**0.34**	**0.10**	**0.58**	**0.93**
Prec	0.11	−0.01	0.22	0.65
Temp	−0.03	−0.13	0.08	0.36
Prec × HSI	−0.05	−0.29	0.19	0.18

The relative importance of the covariate is the sum of AICc weights for the models where the covariate occurs. Random effects for the year and study area are fitted for all models. Coefficients are in bold where CIs do not include zero.

To examine whether the *HSI *× *Date* interaction was consistent between years, we developed the full set of candidate models from a global model including the *HSI *× *Date *× *Year* interaction, with *Year* implemented as a factor and the study area as a random effects variable. Neither models including the interaction *HSI *× *Date *× *Year* (ΔAICc = 31.75) nor *HSI *× *Year* (ΔAICc ≥ 14.48) were retained relative to the best models (ΔAICc < 2). Graphical analyses confirmed that the *HSI *× *Date* interaction was very consistent among years (see online Appendix B3 in Appendix S2).

## 4 Discussion

From an eco‐evolutionary perspective, habitat suitability index (HSI) and demography have strong conceptual links because (1) for a given species, from an evolutionary viewpoint, it is expected that habitat attractiveness will match demographic performance (Battin, 2004; Brambilla & Ficetola, 2012; Hildén, 1965; Losier et al., 2015) and (2) although there are many ecological processes that can influence species’ range patterns and dynamics (Thuiller et al., 2008), these processes affect range dynamics through their effect on demography (reproduction, survival, and dispersal, Cabral & Schurr, 2010; Holt & Keitt, 2005).

However, recent studies indicate that relationships between HSI and demographic components are not always positive (Monnet et al., 2015; Pellissier et al., 2013; Thuiller, Albert, Dubuis, Randin, & Guisan, 2010). This suggests that the ecological processes governing relationships between population demography and a species’ environment may be too complex to be integrated into niche modeling. In parallel, it has also been recently demonstrated that the HSI may be seasonally dynamic (Bateman et al., 2012; Nakazawa et al., 2004; Reside et al., 2010) and that temporal and spatial variations in biotic interactions can affect the results of niche modeling (Wisz et al., 2013). Our study establishes a link between these two categories of results by demonstrating that the relationship between the HSI and demography is itself strongly variable temporally at an intra‐annual scale. One corollary of this is that a static HSI is not sufficient to explain demography.

Our HSI was derived from occurrence data independent from our reproduction data (Monnet et al., 2015). It reflects the propensity of individuals to select various habitat types during the core breeding season. Overall, the Houbara's daily nest survival rate exhibits a strong decrease with the progression of the breeding season (Table 1 and online Appendix B4 in Appendix S2), which is expected with respect to both demographic heterogeneity (e.g., more experienced females tend to lay early in the season and have potentially high breeding success) (Verhulst & Nilsson, 2008), and to meteorological changes along the season, which can also impact nest survival (Verhulst & Nilsson, 2008). Most importantly, nest survival is negatively related to the HSI early in the season, and this relationship becomes weaker with the progression of the season to the extent that it is eventually reversed by the end of the season (Figure 2). Even if this trend presents wide confidence intervals late in the breeding season due to a smaller sample size, the significance of the interaction between *HSI* and *Date* stands as a strong support of the dynamic of the relationship.

It has been suggested that the negative relationships between HSI and demography are related to density‐dependent processes which limit demographic performances at high density (Guisan & Thuiller, 2005; Thuiller et al., 2014). Because of the large spatial scale (75,000 km^2^) and fine temporal scale (intraseasonal changes in relation to daily nest survival) of our study, we could not obtain accurate estimates of Houbara densities along the breeding season and thus could not formally assess the role of density in the demography–HSI relationship. However, studies on habitat suitability generally have reported significant positive correlation between HSI and population densities (Oliver et al., 2012; Tôrres et al., 2012). This is in concordance with the positive correlation observed between autumnal densities of Houbaras and the HSI (ECWP unpublished data, see Appendix B5 in Appendix S2). During the breeding season, Houbaras are nonevenly distributed. In this lekking species, males aggregate to display and female nests are spatially associated to lek occurrence (Hingrat et al. 2004; Hingrat, Saint Jalme, Chalah, Orhant, & Lacroix, 2008) in habitats of highest quality (i.e., with highest HSIs). It is therefore likely that the HSI is positively correlated with Houbara densities during the breeding season and that our results reflect a dynamic balance of the positive effect of highly suitable habitat on nest survival, and the negative effect associated with high Houbara density. Similar observations and interpretations have been obtained for survival probabilities of adult Houbaras with an independent data set (Monnet et al., 2015). Importantly, such relationship between demography and HSI mediated by density‐dependent processes implies that two types of processes occur: (1) Density is higher in highly suitable habitats (high HSIs) (Oliver et al., 2012; Tôrres et al., 2012), and (2) demographic performance is negatively related with density (Brook & Bradshaw, 2006). Both types of relationships are likely to be temporally variable, in turn leading to temporal variation in the HSI–demography relationship.

In Houbara, the first type of process (i.e., dynamic density–HSI relationship) is likely explained by the cyclical switch of the realized niche of Houbara, in which females use different areas in the breeding and wintering seasons (Hingrat, Saint Jalme et al. 2007). At the transition between the breeding and nonbreeding seasons, the expected correlation between the breeding range HSI and actual occurrence (and density) may disappear or be attenuated, in turn reducing any density‐mediated adverse effects on the daily nest survival.

The second type of process (i.e., dynamic relationship between density and demographic rates) is likely related to the dynamics of biotic interactions. During the breeding season, females nesting mostly at the periphery of leks might undergo high levels of male harassment, in turn affecting negatively the time they allocate in feeding trips and in attending the nest (Stone, 1995) or even increasing their detectability to predators (Magurran & Nowak, 1991). Additionally, the high breeding activity and higher Houbara density in high HSI sites can attract more predators (see, e.g., Keyser, Hill, & Soehren, 1998; Larivière & Messier, 1998), inducing higher rates of nest predation (Gunnarsson & Elmberg, 2008; Vickery, Hunter, & Wells, 1992). Observed changes in the nest survival–HSI relationship are consistent with the expected reduction in these antagonist effects along the season. The male displaying season ends sooner than the nesting season (Hingrat, Saint Jalme et al. 2007), meaning females will face lower levels of male harassment toward the end of the season. The decreasing number of nesting females will also reduce competition for nest sites and food resources. Furthermore, Red Fox (*Vulpes vulpes.),* the main predators of Houbara nests (Bacon & Hingrat, unpublished data), are expected to change their foraging behavior along the season (Cavallini & Lovari, 1991) in relation to the dynamic abundance of alternative resources such as arthropods (Hingrat, Ysnel et al. 2007) or even bird chicks (rather than eggs).

With the exception of Pellissier et al. (2013), who found a significant relationship between nest success and HSI in the Ruddy Turnstone (*Arenaria interpres*) across years in relation to snow cover, we are not aware of other characterizations of the dynamics of the link between HSI and a demographic parameter. Our results regarding the Houbara indicate that this link can be variable within year, probably in relation to the dynamics of intra‐ and interspecific interactions and/or habitat selection, but with a similar pattern between years. Niche modeling has become an increasingly widely used technique in ecology, having been successfully used to address a large range of fundamental and applied research questions (Monnet, Hingrat, & Jiguet, 2015; Yannic et al., 2014). Here, we used a HSI computed over a period covering the core breeding season of Houbaras using powerful analytical techniques (Thuiller et al., 2009). However, in spite of its sophistication, our static HSI does not appear to reflect the complexity of demographic patterns and their intraseasonal dynamics, and using it as a mere proxy for demography would lead to incorrect inferences. Other analytical tools than niche modeling exist to study species distribution, such as the resource selection function (Manly, McDonald, Thomas, McDonald, & Erickson, 2002). Although these tools have been used to study demography (McLoughlin, Boyce, Coulson, & Clutton‐Brock, 2006; McLoughlin et al., 2007), it appears that the same limitations occur as for niche modeling (Boyce, Vernier, Nielsen, & Schmiegelow, 2002; McLoughlin, Morris, Fortin, Vander Wal, & Contasti, 2010). In this context, advances in the development of dynamic habitat suitability metrics (Yannic et al., 2014) and the inclusion of dynamic biotic interactions in niche modeling (Wisz et al., 2013) offer promising new approaches to understand the link between HSI and demography. Three important implications of these approaches are the appreciation of the ecological processes that shape species'ranges (Bateman et al., 2012; Thuiller et al., 2008), the understanding of the evolutionary processes driving habitat selection (Battin, 2004; Jones, 2001), and the development of mechanistic models of species distribution (i.e., models describing all important processes potentially constraining a species’ range) to provide more accurate predictions than the widely used correlative models (niche modeling) (Buckley et al., 2010; Kearney, 2006; Kearney & Porter, 2009).

## Conflict of Interest

None declared.
