# Supplementary material for: Habitat suitability and demography, a time‐dependent relationship
Source: Ecol Evol. 2017 Mar 2;7(7):2214–22. doi: 10.1002/ece3.2821 (PMC5383465; doi:10.1002/ece3.2821)
Supplement: Supplementary file 1 [file ECE3-7-2214-s001.docx]

**Online Supplementary Materials**

*Online Appendix S1: Methods*

Online Appendix A1: Number of nests monitored per year

Table A1.1. Nest visit intervals distribution statistics in function of the year and the nest location technique (Transmitter = nest located from females fitted with VHF or satellite transmitters, Shepherd = nests located from a collaboration with local shepherd).

|  | | Visit intervals | | | | | | | | |  |
| --- | --- | --- | --- | --- | --- | --- | --- | --- | --- | --- | --- |
| Year | Nest location technique | | Mean | Median | Standard deviation | Standard error | 1st centile | 9th centile | N nests | N visit intervals | |
| 2003 | Transmitter | | 7.45 | 7.00 | 4.67 | 0.65 | 2.00 | 14.00 | 24 | 51 | |
| 2003 | Shepherd | | 7.39 | 7.00 | 4.73 | 0.58 | 1.00 | 12.00 | 35 | 67 | |
| 2004 | Transmitter | | 9.36 | 8.00 | 5.28 | 0.47 | 4.00 | 15.00 | 68 | 124 | |
| 2004 | Shepherd | | 8.60 | 7.00 | 5.85 | 0.39 | 1.00 | 17.00 | 108 | 220 | |
| 2005 | Transmitter | | 5.20 | 5.00 | 2.37 | 0.43 | 2.00 | 8.10 | 12 | 30 | |
| 2005 | Shepherd | | 5.72 | 5.00 | 3.90 | 0.72 | 1.80 | 11.20 | 14 | 29 | |
| 2006 | Transmitter | | 6.74 | 7.00 | 3.03 | 0.25 | 2.00 | 10.00 | 61 | 149 | |
| 2006 | Shepherd | | 5.13 | 4.00 | 4.25 | 0.16 | 1.00 | 10.00 | 236 | 728 | |
| 2007 | Transmitter | | 5.07 | 5.00 | 2.54 | 0.19 | 2.00 | 8.00 | 59 | 182 | |
| 2007 | Shepherd | | 5.74 | 5.00 | 4.65 | 0.34 | 1.00 | 13.00 | 82 | 192 | |
| 2008 | Transmitter | | 5.24 | 5.00 | 2.81 | 0.18 | 2.00 | 8.00 | 75 | 238 | |
| 2008 | Shepherd | | 5.29 | 5.00 | 4.16 | 0.41 | 1.00 | 10.00 | 43 | 101 | |
| 2009 | Transmitter | | 5.02 | 5.00 | 2.26 | 0.21 | 2.00 | 7.00 | 37 | 115 | |
| 2009 | Shepherd | | 5.42 | 5.00 | 4.28 | 0.26 | 1.00 | 12.00 | 100 | 262 | |
| 2010 | Transmitter | | 5.50 | 5.00 | 2.29 | 0.23 | 2.00 | 8.00 | 38 | 103 | |
| 2010 | Shepherd | | 5.06 | 5.00 | 3.35 | 0.19 | 1.00 | 9.00 | 118 | 325 | |
| 2011 | Transmitter | | 5.71 | 6.00 | 2.35 | 0.48 | 1.60 | 8.00 | 11 | 24 | |
| 2011 | Shepherd | | 5.44 | 5.00 | 3.77 | 0.14 | 1.00 | 9.50 | 287 | 716 | |
| 2012 | Transmitter | | 6.05 | 6.00 | 2.47 | 0.31 | 3.00 | 8.00 | 23 | 62 | |
| 2012 | Shepherd | | 5.19 | 5.00 | 3.30 | 0.08 | 1.00 | 9.00 | 605 | 1713 | |
| 2013 | Transmitter | | 6.24 | 7.00 | 3.16 | 0.47 | 2.00 | 9.00 | 21 | 46 | |
| 2013 | Shepherd | | 6.26 | 6.00 | 4.34 | 0.11 | 1.00 | 12.00 | 574 | 1494 | |
| 2014 | Transmitter | | 5.16 | 6.00 | 2.21 | 0.44 | 2.00 | 7.00 | 7 | 25 | |
| 2014 | Shepherd | | 5.38 | 5.00 | 3.55 | 0.10 | 1.00 | 9.80 | 470 | 1333 | |


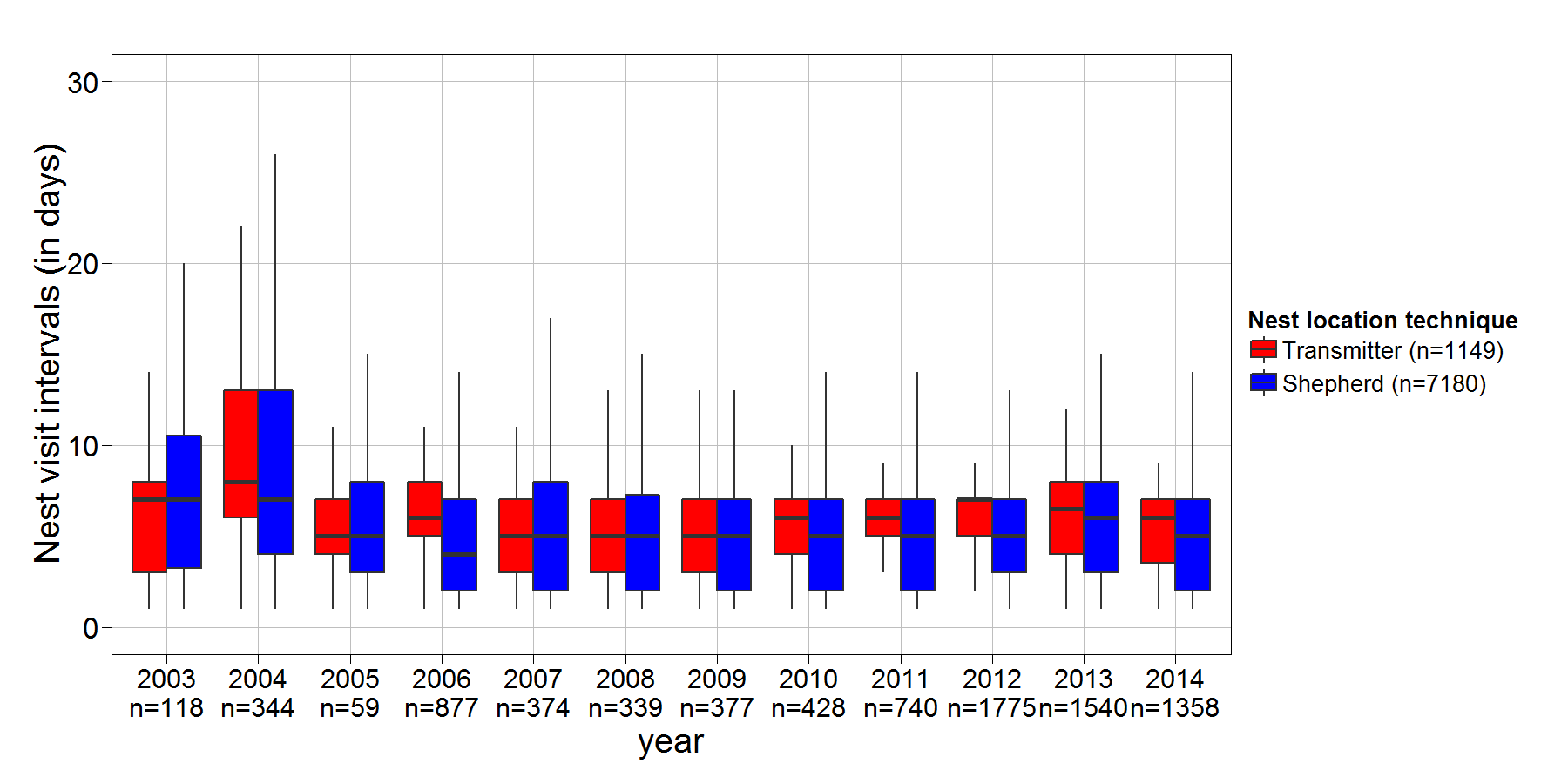


FigureA1.1. Nest visit intervals distribution (boxplot) in function of the year and the nest location technique (Transmitter = in red, nest located from females fitted with VHF or satellite transmitters, Shepherd = in blue, nests located from a collaboration with local shepherd).

TableA1.2. Nest visit intervals distribution statistics in function of the year and the nest monitoring technique (Presence or not of camera trap).

|  | | Visit intervals | | | | | | | | |  |
| --- | --- | --- | --- | --- | --- | --- | --- | --- | --- | --- | --- |
| Year | Presence camera trap | | Mean | Median | Standard deviation | Standard error | 1st centile | 9th centile | N nests | N visit intervals | |
| 2003 | No | | 7.42 | 7.00 | 4.68 | 0.43 | 2.00 | 13.00 | 59 | 118 | |
| 2004 | No | | 8.88 | 8.00 | 5.66 | 0.31 | 2.00 | 15.00 | 176 | 344 | |
| 2005 | No | | 5.46 | 5.00 | 3.20 | 0.42 | 2.00 | 10.00 | 26 | 59 | |
| 2006 | No | | 5.40 | 5.00 | 4.11 | 0.14 | 1.00 | 10.00 | 297 | 877 | |
| 2007 | No | | 5.41 | 5.00 | 3.78 | 0.20 | 1.00 | 10.00 | 141 | 374 | |
| 2008 | No | | 5.25 | 5.00 | 3.27 | 0.18 | 2.00 | 9.00 | 118 | 339 | |
| 2009 | No | | 5.29 | 5.00 | 3.78 | 0.19 | 1.00 | 9.00 | 137 | 377 | |
| 2010 | No | | 5.17 | 5.00 | 3.13 | 0.15 | 1.00 | 9.00 | 156 | 428 | |
| 2011 | No | | 5.45 | 5.00 | 3.73 | 0.14 | 1.00 | 9.00 | 298 | 740 | |
| 2012 | No | | 5.22 | 5.00 | 3.27 | 0.08 | 1.00 | 9.00 | 628 | 1775 | |
| 2013 | No | | 6.24 | 6.00 | 4.43 | 0.12 | 1.00 | 13.00 | 595 | 1408 | |
| 2013 | Yes | | 6.41 | 6.00 | 2.65 | 0.23 | 3.00 | 10.00 | 116 | 132 | |
| 2014 | No | | 5.16 | 5.00 | 3.68 | 0.11 | 1.00 | 10.00 | 477 | 1172 | |
| 2014 | Yes | | 6.70 | 7.00 | 1.90 | 0.14 | 4.00 | 8.00 | 115 | 186 | |


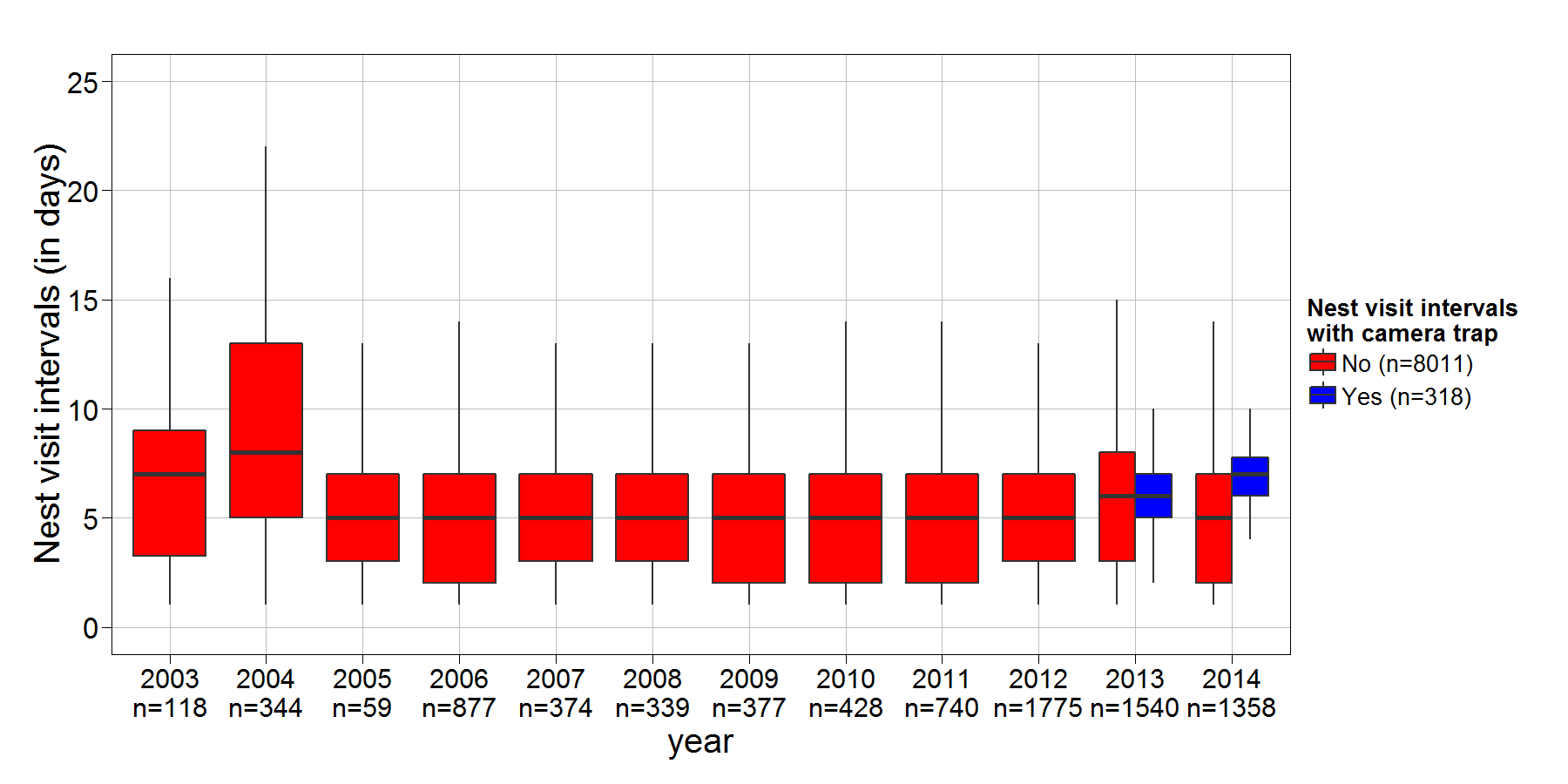


FigureA1.2. Nest visit intervals distribution (boxplot) in function of the year and the nest monitoring technique (Presence (in blue) or not (in red) of camera trap).

Online Appendix A2: Environmental data for species distribution modelling (Monnet *et al*. 2015)

Monnet *et al.* (2015) used both climate and land cover variables for species distribution modelling: the three first axes of a principal components analysis calibrated on eight bioclimatic variables (Table A1) and seven land cover variables (Table A2).

Table A2.1. Bioclimatic variables from the Worldclim database (Hijmas *et al.* 2005).

| Code | Variables |
| --- | --- |
| BIO1 | mean annual temperature |
| BIO6 | minimum temperature of the coldest month |
| BIO5 | maximum temperature of the warmest month |
| BIO4 | temperature seasonality (standard deviations of mean monthly temperatures) |
| BIO12 | annual precipitation |
| BIO14 | precipitation of the driest month |
| BIO13 | precipitation of the wettest month |
| BIO15 | precipitation seasonality (coefficient of variation of monthly precipitations) |

Table A2.2. Summarized land cover variables from global land-cover classes (Mayaux *et al.* 2004).

| Global land-cover classes | Summarized land cover variables |
| --- | --- |
| Open deciduous shrub land | open shrubland |
| Sparse grassland | sparse grassland |
| Croplands (>50%) | cropland |
| Irrigated croplands | cropland |
| Tree crops | cropland |
| Bare rock | bare rock |
| Stony desert | stony desert |
| Sandy desert and dunes | sandy desert |
| Closed evergreen lowland forest | unfavorable |
| Degraded evergreen lowland forest | unfavorable |
| Montane evergreen forest (> 1500 m) | unfavorable |
| Sub-montane forest (>900 m) | unfavorable |
| Mangrove | unfavorable |
| Mosaic forest / croplands | unfavorable |
| Closed deciduous forest (Miombo) | unfavorable |
| Deciduous woodland | unfavorable |
| Salt hardpans | unfavorable |
| Waterbodies | unfavorable |
| Cities | unfavorable |

Online References for Appendix S1

Hijmans RJ, Camera SE, Parra JL, Jones PG, Jarvis A (2005). Very high resolution interpolated climate surfaces for global land areas. Int. J. Climatol. 25: 1965 – 1978. doi:10.1002/joc.1276

Mayaux P, Bartholomé E, Fritz S, Belward A (2004). A new land-cover map of Africa for the year 2000. J. Biogeogr. 31: 861 – 877. doi:10.1111/j.1365-2699.2004.01073.x

Monnet A-C, Hardouin LA, Robert A, Hingrat Y, Jiguet F (2015). Evidence of a link between demographic rates and species habitat suitability from post release movements in a reinforced bird population. Oikos 124: 1089–1097 doi:10.1111/oik.01834

*Online Appendix S2: Results*

Online Appendix B1: Habitat suitability index (HSI) distribution at nests locations


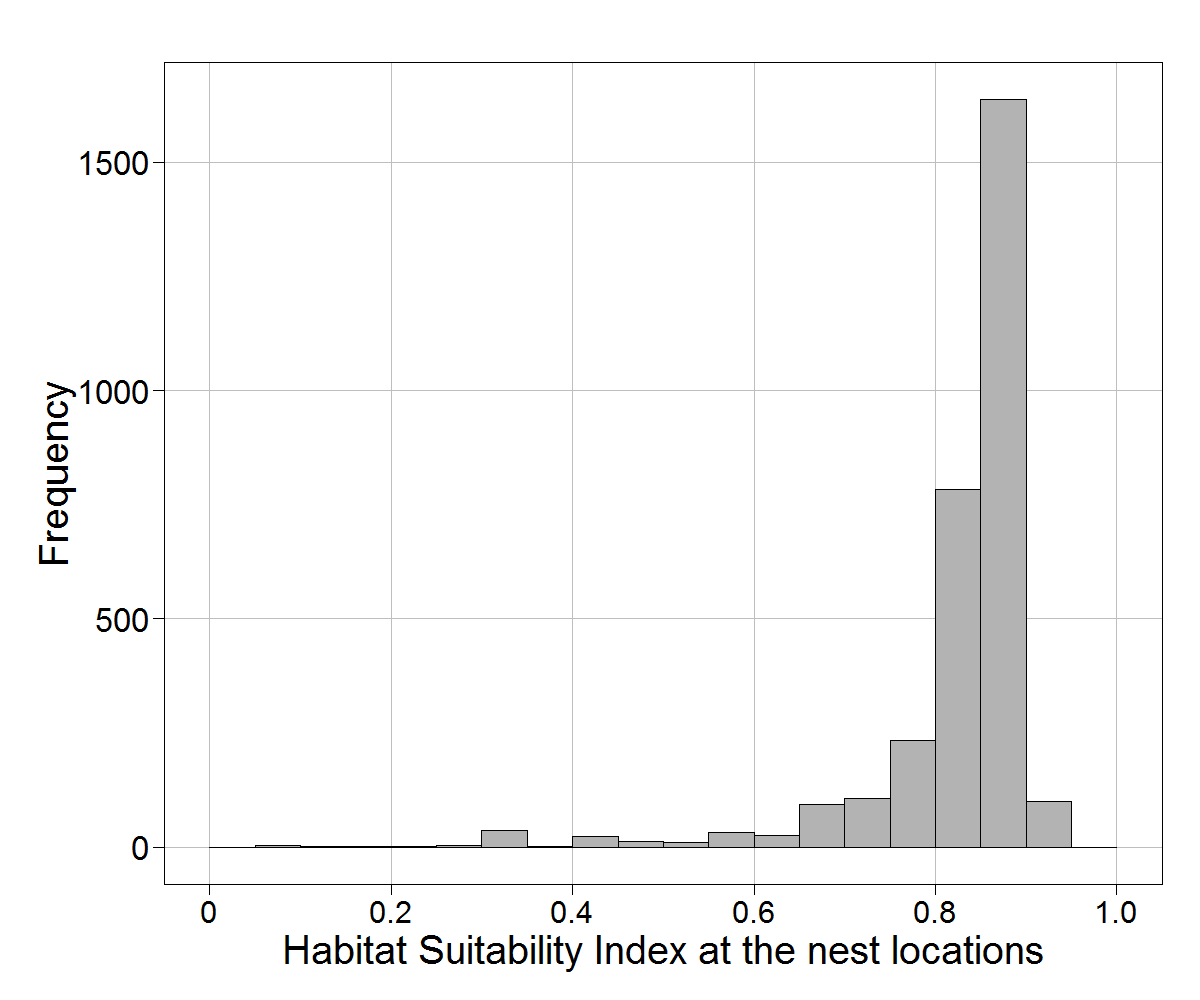


Figure B1. Frequency of the Habitat Suitability Index (HSI) at the nests locations

Online appendix B2 : Multi-model inference – Model selection

TableB2.1. Model selection from candidates model including combinations of additive effects (Step1). Date = Date within the breeding season, Prec = Average precipitation from the year preceding the breeding season, Temp = Averaged temperature from the year preceding the breeding season, HSI = Habitat suitability index at the nest location. Blank spaces indicate covariates not included in the model. df = degree of freedom, logLik = log likelhood.

| Date | Prec | Temp | HSI | df | logLik | AICc | ΔAICc | AICc weight |
| --- | --- | --- | --- | --- | --- | --- | --- | --- |
| -0.38 | 0.09 |  | -0.11 | 6 | -3457.78 | 6927.60 | 0.00 | 0.25 |
| -0.38 |  |  | -0.11 | 5 | -3458.82 | 6927.70 | 0.08 | 0.24 |
| -0.38 | 0.09 |  |  | 5 | -3459.54 | 6929.10 | 1.52 | 0.12 |
| -0.38 | 0.10 | -0.03 | -0.11 | 7 | -3457.63 | 6929.30 | 1.69 | 0.11 |
| -0.38 |  |  |  | 4 | -3460.63 | 6929.30 | 1.70 | 0.11 |
| -0.38 |  | -0.01 | -0.11 | 6 | -3458.81 | 6929.60 | 2.07 | 0.09 |
| -0.38 | 0.09 | -0.02 |  | 6 | -3459.48 | 6931.00 | 3.41 | 0.05 |
| -0.38 |  | 0.00 |  | 5 | -3460.63 | 6931.30 | 3.70 | 0.04 |
|  |  |  | -0.11 | 4 | -3481.04 | 6970.10 | 42.52 | 0.00 |
|  |  |  |  | 3 | -3482.96 | 6971.90 | 44.34 | 0.00 |
|  |  | -0.01 | -0.11 | 5 | -3481.01 | 6972.00 | 44.45 | 0.00 |
|  | 0.01 |  | -0.11 | 5 | -3481.03 | 6972.10 | 44.50 | 0.00 |
|  | 0.00 |  |  | 4 | -3482.95 | 6973.90 | 46.34 | 0.00 |
|  |  | 0.00 |  | 4 | -3482.95 | 6973.90 | 46.34 | 0.00 |
|  | 0.01 | -0.01 | -0.12 | 6 | -3481.00 | 6974.00 | 46.43 | 0.00 |
|  | 0.00 | 0.00 |  | 5 | -3482.95 | 6975.90 | 48.34 | 0.00 |

TableB2.2. Model selection from candidates model including combinations of additive effects and interaction terms (step 2). Date = Date within the breeding season, Prec = Average precipitation from the year preciding the breeding season, Temp = Averaged temperature from the year preceding the breeding season, HSI = Habitat suitability index at the nest location. Blank spaces indicate covariates and interactions not included in the model. df = degree of freedom, logLik = log likelhood.

| Date | Prec | Temp | HSI | Date x HSI | Prec x HSI | Temp x HSI | df | logLik | AICc | ΔAICc | AICc wieght |
| --- | --- | --- | --- | --- | --- | --- | --- | --- | --- | --- | --- |
| -0.34 | 0.11 |  | -0.18 | 0.34 |  |  | 7 | -3453.77 | 6921.50 | 0.00 | 0.28 |
| -0.35 |  |  | -0.18 | 0.33 |  |  | 6 | -3455.04 | 6922.10 | 0.55 | 0.21 |
| -0.35 | 0.11 | -0.03 | -0.18 | 0.34 |  |  | 8 | -3453.61 | 6923.20 | 1.70 | 0.12 |
| -0.34 | 0.10 |  | -0.17 | 0.36 | -0.05 |  | 8 | -3453.70 | 6923.40 | 1.86 | 0.11 |
| -0.35 |  | 0.00 | -0.18 | 0.33 |  |  | 7 | -3455.04 | 6924.10 | 2.56 | 0.08 |
| -0.35 | 0.11 | -0.02 | -0.19 | 0.35 |  | 0.06 | 9 | -3453.54 | 6925.10 | 3.56 | 0.05 |
| -0.35 | 0.10 | -0.03 | -0.18 | 0.36 | -0.04 |  | 9 | -3453.55 | 6925.10 | 3.59 | 0.05 |
| -0.34 |  | 0.00 | -0.18 | 0.33 |  | 0.07 | 8 | -3454.94 | 6925.90 | 4.35 | 0.03 |
| -0.34 | 0.10 | -0.02 | -0.18 | 0.36 | -0.03 | 0.05 | 10 | -3453.51 | 6927.00 | 5.49 | 0.02 |
| -0.38 | 0.09 |  | -0.11 |  |  |  | 6 | -3457.78 | 6927.60 | 6.03 | 0.01 |
| -0.38 |  |  | -0.11 |  |  |  | 5 | -3458.82 | 6927.70 | 6.11 | 0.01 |
| -0.38 | 0.11 |  | -0.12 |  | 0.09 |  | 7 | -3457.48 | 6929.00 | 7.42 | 0.01 |
| -0.38 | 0.09 |  |  |  |  |  | 5 | -3459.54 | 6929.10 | 7.55 | 0.01 |
| -0.38 | 0.10 | -0.03 | -0.11 |  |  |  | 7 | -3457.63 | 6929.30 | 7.72 | 0.01 |
| -0.38 |  |  |  |  |  |  | 4 | -3460.63 | 6929.30 | 7.72 | 0.01 |
| -0.38 |  | -0.01 | -0.11 |  |  |  | 6 | -3458.81 | 6929.60 | 8.09 | 0.01 |
| -0.38 | 0.11 | -0.03 | -0.12 |  | 0.09 |  | 8 | -3457.29 | 6930.60 | 9.05 | 0.00 |
| -0.38 | 0.09 | -0.02 |  |  |  |  | 6 | -3459.48 | 6931.00 | 9.43 | 0.00 |
| -0.38 | 0.10 | -0.03 | -0.11 |  |  | 0.03 | 8 | -3457.61 | 6931.20 | 9.69 | 0.00 |
| -0.38 |  | 0.00 |  |  |  |  | 5 | -3460.63 | 6931.30 | 9.72 | 0.00 |
| -0.38 |  | -0.01 | -0.11 |  |  | 0.04 | 7 | -3458.78 | 6931.60 | 10.03 | 0.00 |
| -0.38 | 0.11 | -0.03 | -0.13 |  | 0.11 | 0.07 | 9 | -3457.20 | 6932.40 | 10.89 | 0.00 |
|  |  |  | -0.11 |  |  |  | 4 | -3481.04 | 6970.10 | 48.55 | 0.00 |
|  |  |  |  |  |  |  | 3 | -3482.96 | 6971.90 | 50.37 | 0.00 |
|  |  | -0.01 | -0.11 |  |  |  | 5 | -3481.01 | 6972.00 | 50.48 | 0.00 |
|  | 0.01 |  | -0.11 |  |  |  | 5 | -3481.03 | 6972.10 | 50.52 | 0.00 |
|  | 0.03 |  | -0.13 |  | 0.11 |  | 6 | -3480.54 | 6973.10 | 51.54 | 0.00 |
|  |  | -0.01 | -0.12 |  |  | 0.08 | 6 | -3480.85 | 6973.70 | 52.17 | 0.00 |
|  | 0.00 |  |  |  |  |  | 4 | -3482.95 | 6973.90 | 52.37 | 0.00 |
|  |  | 0.00 |  |  |  |  | 4 | -3482.95 | 6973.90 | 52.37 | 0.00 |
|  | 0.01 | -0.01 | -0.12 |  |  |  | 6 | -3481.00 | 6974.00 | 52.46 | 0.00 |
|  | 0.03 | -0.02 | -0.13 |  | 0.12 |  | 7 | -3480.48 | 6975.00 | 53.43 | 0.00 |
|  | 0.01 | -0.01 | -0.12 |  |  | 0.08 | 7 | -3480.84 | 6975.70 | 54.16 | 0.00 |
|  | 0.00 | 0.00 |  |  |  |  | 5 | -3482.95 | 6975.90 | 54.37 | 0.00 |
|  | 0.03 | -0.01 | -0.14 |  | 0.15 | 0.14 | 8 | -3480.08 | 6976.20 | 54.62 | 0.00 |

Online Appendix B3: Consistency of HSI × Date among years


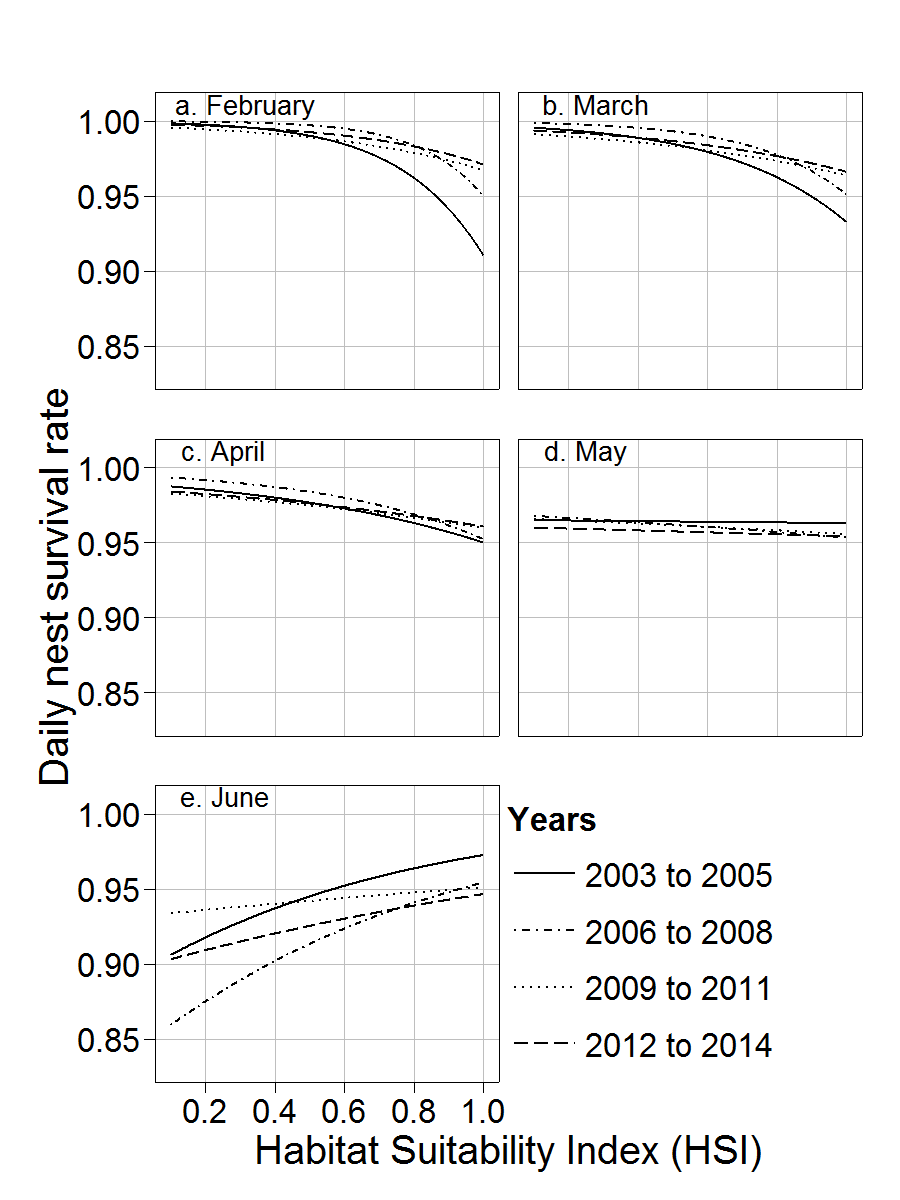


Figure B3. Relationship between the daily nest survival rate and the Habitat Suitability Index (HSI), with inter-annual grouping for each month of the breeding season. a. February, b. March, c. April, d. May and e. June.

Online Appendix B4: Daily nest survival rate (± 95% CI) along the breeding season for various values of Habitat Suitability Index (HSI).


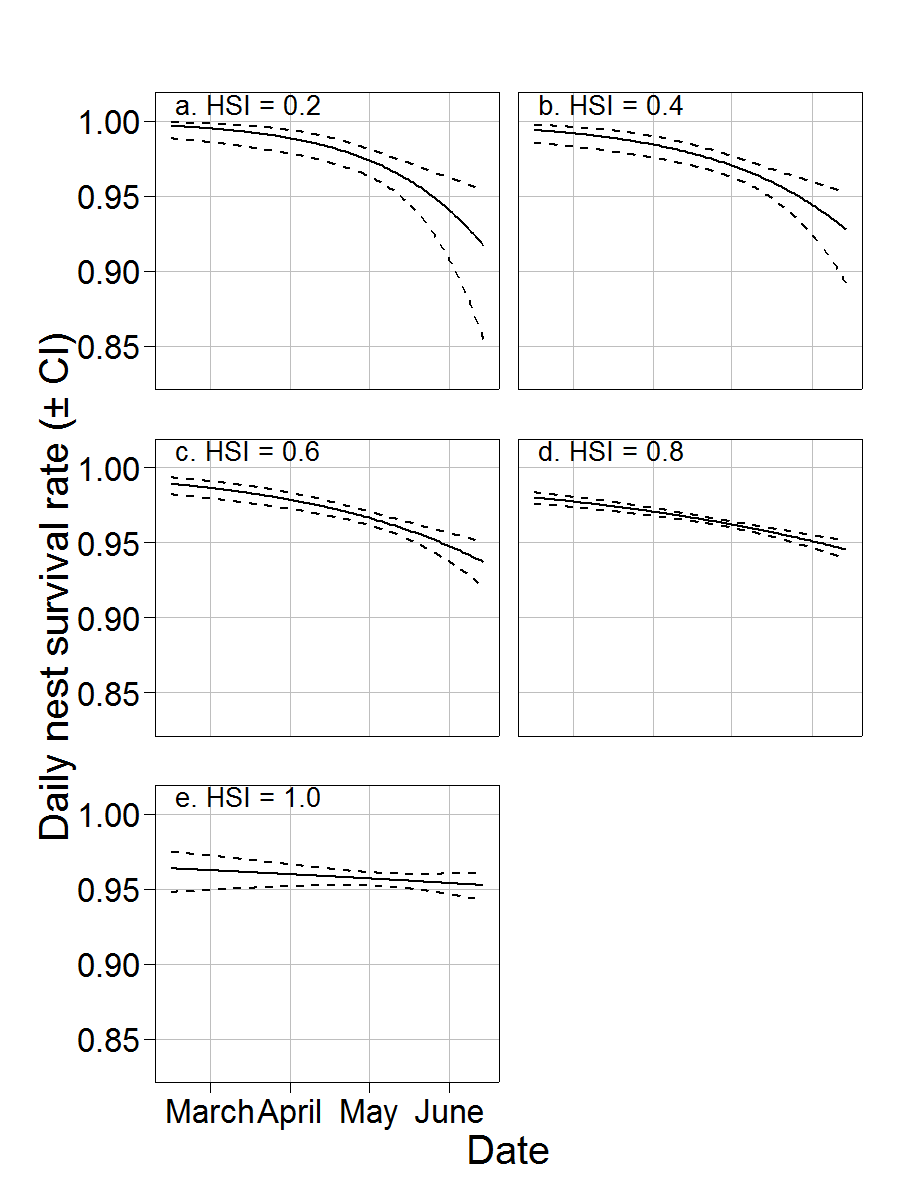


Figure B4. Relationship between the daily nest survival rate (± 95% CI) and the date within the breeding season for different values of Habitat Suitability Index (HSI). a. HSI = 0.2, b. HSI=0.4, c. HSI=0.6, d HSI=0.8, and e. HSI=1.0.

Online Appendix B5: North African Houbara Bustard (*Chlamydotis undulata undulata*) autum densities in function of intervals of Habitat Suitability Index (HSI).


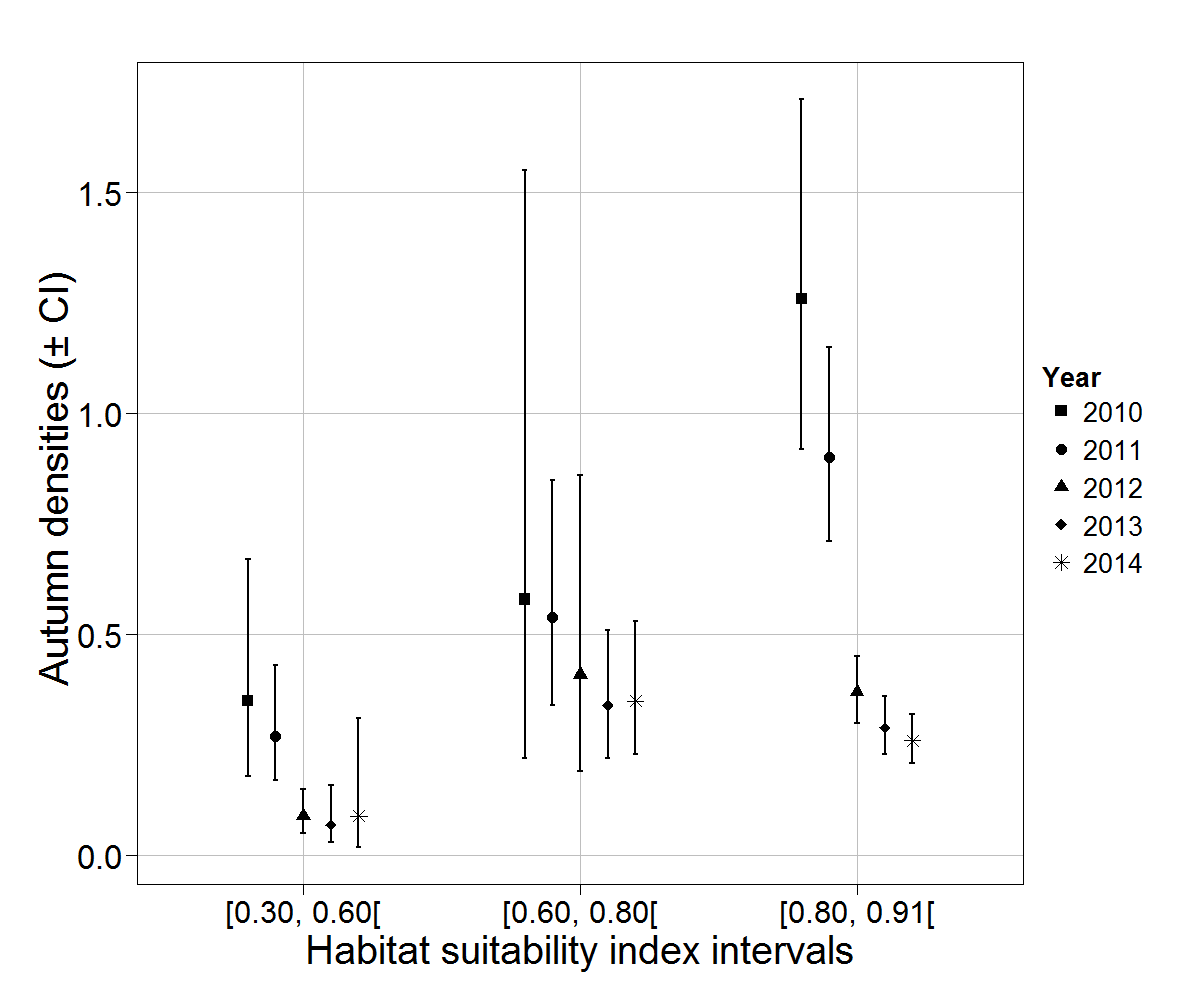


Figure B5. North African Houbara bustard (*Chlamydotis undulata undulata*) autum densities (± 95% confidence intervals) in function of Habitat Suitability Index intervals.
